# Registered nurses’ knowledge and practice of preoperative fasting and medication administration

**DOI:** 10.4102/hsag.v29i0.2490

**Published:** 2024-02-16

**Authors:** Justin C. King, Adele de Goede, Janet Bell

**Affiliations:** 1 Department of Anaesthesiology and Critical Care, Faculty of Medicine and Health Sciences, Stellenbosch University, Cape Town, South Africa; 2 Department of Nursing and Midwifery, Faculty of Medicine and Health Sciences, Stellenbosch University, Cape Town, South Africa

**Keywords:** nursing knowledge, nil per os times, medication administration, tertiary hospital, South Africa

## Abstract

**Background:**

Knowledge of fasting or Nil Per Os (NPO) guidelines is an essential component of nursing care in the preoperative period.

**Aim:**

To describe registered nurses’ (RNs) knowledge and management of the preoperative NPO period.

**Setting:**

Selected surgical wards in a tertiary hospital in the Western Cape, South Africa.

**Methods:**

Quantitative descriptive, cross-sectional study utilising a structured questionnaire. The population consisted of RNs working in selected surgical wards. Convenience sampling was used and adequate knowledge was determined as ≥ 90%.

**Results:**

The response rate was 100%. Of the 68 participants, 48 (70.6%) held a diploma and 20 (29.4%) held a degree as the highest academic qualification achieved. Sixty-one (89.7%) participants knew the correct reason for keeping patients NPO. Sixty-five (95.6%) knew the correct answer for the NPO time for solids while only 27 (39.7%) knew the correct answer for clear fluids. Only 30 (44.1%), 26 (38.2%) and 33 (48.5%) participants, respectively, answered the questions about oral analgesia, oral antibiotics and chronic medication administration during the NPO period correctly. Significantly more degree participants knew the correct answer for the fasting time for non-human milk (*p* = 0.005) and more diploma participants would administer chronic medication during the NPO period (*p* = 0.037).

**Conclusion:**

Inadequate knowledge of NPO times for various fluids and unsatisfactory practice of medication administration for oral and chronic medication require attention.

**Contribution:**

This study highlights the importance that ongoing education is needed to ensure that patients receive the most up-to-date evidence-based care during the NPO period.

## Introduction

Preoperative fasting or Nil Per Os (NPO) is defined as ‘the restriction of food and fluid intake prior to general anaesthesia or procedural sedation’ and is imperative for ensuring patient safety (Brady 2003). Induction of anaesthesia or sedation results in the inhibition of the gag, cough and swallow reflexes that normally protect the airway, thereby increasing the risk of pulmonary aspiration should regurgitation or vomiting occur (Brady 2003). This potentially devastating complication was first described in 1946 by Mendelson (1946:191). Fasting requirements are prescribed by doctors but generally managed by nurses and carried out in collaboration with patients.

The 2017 American Society of Anesthesiologists fasting guidelines (American Society of Anesthesiologists 2017:376) have been endorsed by most common interest groups, including the South African Society of Anaesthesiologists (SASA 2018:1). The guidelines recommend fasting times of 8 h for fatty foods, 6 h for light meals, non-human milk and infant formula, 4 h for breast milk and 2 h for clear fluids. The only nursing fasting guidelines that were identified were from the Royal College of Nursing in the United Kingdom. The guidelines state a minimum fasting period of 6 h for food and 2 h for clear fluids (Royal College of Nursing 2005). Fasting guidelines are applicable prior to a procedure where some form of general anaesthesia, regional anaesthesia or sedation is used. Despite the availability of guidelines, many patients awaiting surgery in South African public institutions are kept NPO for longer than necessary (Lamacraft et al. 2017:910).

Prolonged fasting may potentially result in preventable physiological and psychological complications (De Aguilar-Nascimento & Dock-Nascimento 2010:57). These complications include, but are not limited to, anxiety, thirst, hunger, metabolic derangement, nausea and vomiting. A cross-sectional survey conducted in 2011 by Tosun, Yava and Açikel (2015:156) in Turkey assessed the effects of perioperative fasting and fluid limitation in 99 patients undergoing elective laparoscopic cholecystectomy. The mean preoperative fasting time for solids was 14.70 (±3.14) hours and for fluids 11.25 (±3.74) hours, causing nearly 59% of patients to experience moderate anxiety. Patients fasting for 12 h or longer had higher hunger, thirst, nausea and pain scores (Tosun et al. 2015:156). A 2012 qualitative Australian study showed that thirst during the fasting period was reported as the worst physical effect of prolonged fasting (Carey, Conchin & Bloomfield-Stone 2015:1946). A retrospective study conducted between November 2013 and February 2016 in Torbay Day Hospital in the United Kingdom examined the incidence of postoperative nausea and vomiting before and after the change to unrestricted preoperative clear oral fluids. The authors found that the rates of nausea within 24 h postoperatively were 5.2% in patients not allowed to drink from 2 h prior to surgery and 3.8% in patients allowed to drink up until the time of surgery. The corresponding rates of vomiting were 2.8% with restricted fluid intake and 2.2% with unrestricted fluid intake. This study suggested that the liberal consumption of clear fluids before anaesthesia reduced postoperative nausea and vomiting (McCracken & Montgomery 2018:337).

In the 2014 Guideline on Perioperative Cardiovascular Evaluation and Management of Patients Undergoing Non-Cardiac Surgery from the American College of Cardiology and the American Heart Association (Fleisher et al. 2014:278), recommendations are made for the administration of chronic medication in cardiac patients undergoing non-cardiac surgery. The 2002 National Confidential Enquiry into Perioperative Deaths Report (National Confidential Enquiry into Perioperative Deaths [NCEPOD] 2002) found that between 20% and 40% of patients were not given essential regular medication before procedures. A review of patients’ drug charts showed the reason for patients not receiving their essential medication was because they were classified as NPO for the perioperative period (National Enquiry into Perioperative Deaths [NCEPOD] 2002). There is a paucity in South African literature regarding the administration of chronic and other prescribed medication to patients by nursing personnel during NPO periods.

As a result of high volumes and staffing constraints, not all patients waiting for emergency surgery in South African public institutions are assessed preoperatively in the ward by an anaesthetist but rather assessed by surgical doctors. Nurses working in the surgical wards play an important and mostly overlooked role in the daily management and preparation of these patients for surgery.

## Aim

This study aimed to describe registered nurses’ (RNs) knowledge and management of the preoperative NPO period in selected surgical wards at Tygerberg Academic Hospital (TBH). The primary objectives of this study were to describe the RNs knowledge of the reason for being NPO, the preoperative fasting times for various foods and fluids and the practices of medication administration during the NPO period. The secondary objective was to compare knowledge and practice between diploma and degree nurses.

## Research design and methods

### Design

This was a quantitative descriptive, cross-sectional study utilising a structured questionnaire.

### Setting

The study was conducted at TBH in the Western Cape, South Africa (SA). This tertiary institution, affiliated to Stellenbosch University, is the largest public hospital in the Western Cape and the second largest public hospital in SA, with a bed occupancy of 1384. Surgical wards included general surgical and specialised surgical disciplines. These disciplines included orthopaedics, gynaecology, burns, otorhinolaryngology and paediatric surgery.

### Study population and sampling strategy

The study population included RNs working in selected surgical wards at TBH. An RN in SA is defined as ‘a person registered as a nurse or midwife in terms of the Nursing Act (No. 33 of 2005)’ (Republic of South Africa Nursing Act 2005). An RN in SA may be registered as a professional nurse either via a diploma or a degree programme, the difference being the academic level at which their studies are completed (Republic of South Africa Nursing Act 2005). A biostatistician from the Department of Epidemiology and Biostatistics at Stellenbosch University was consulted to calculate the sample size. With the help of nursing management, a study population of 133 RNs was calculated by identifying each ward that would be included in the study and the total number of RNs currently employed in each ward. A sample size of 68 out of 133 RNs was estimated to allow a precision of 5% (half-width of the 95% confidence interval to estimate the true percentage). A sample size of 68 from a population of 133 produced a two-sided 95% confidence interval with a precision of 0.0500 when the actual proportion was near 0.9000. Convenience sampling was employed.

### Data collection, validity and reliability

No published questionnaire suitable for this study could be identified. Following a review of the literature and reviewing preoperative medication administration during the NPO period, a draft questionnaire was developed, enhancing content validity. The draft questionnaire was reviewed by 15 anaesthesiologists in the Department of Anaesthesiology and Critical Care and a senior nursing educator from the Department of Nursing and Midwifery at Stellenbosch University and their suggestions were incorporated, enhancing content and face validity. The draft questionnaire was then piloted in a TBH surgical ward not included in the study. Ten RNs participated, and their suggestions were incorporated, contributing to face validity.

The final anonymous questionnaire consisted of 20 questions, including four demographic questions. Section A, with seven questions, examined the reason for fasting and the fasting times for foods and fluids. Section B, with nine questions, examined the current practice of medication administration and permission to sip water. Correct answers for knowledge of fasting times were based on the SASA 2020 Guidelines for the Safe Use of Procedural Sedation and Analgesia for Diagnostic and Therapeutic Procedures in Adults (Roelofse & Jansen van Rensburg 2020:1). Correct practice of medication administration during the NPO period was based on the 2014 Guideline on Perioperative Cardiovascular Evaluation and Management of Patients Undergoing Non-Cardiac Surgery from the American College of Cardiology and the American Heart Association (Fleisher et al. 2014:278). Correct practice was classified as medication that should still be administered during the NPO period. A panel of experts in anaesthesia determined adequate knowledge and practice as ≥ 90%.

The questionnaires were distributed to participants once informed consent was obtained. Questionnaires were available in English, Afrikaans and isiXhosa. The completion of the questionnaire was carried out at the convenience of the participants, and estimated time for completion was between 5 and 10 min. This ensured that there was minimal disruption and interference with allocated duties and patient care. Each questionnaire handed out was placed in an envelope that was numbered from 1 to 68, and upon collection of the questionnaires, the number on each sealed envelope was cross-checked on a separate sheet. This ensured a track record of outstanding questionnaires and the total number of returned questionnaires. The Informal Nurse Training Unit (INTU), which is responsible for the co-ordination of research relating to nursing services at TBH, assisted with the distribution and collection of questionnaires. Data collection was performed over 1 week in August 2021 during specifically identified times of the day, incorporating both day and night staff members as well as staff from alternating shifts. Strict coronavirus disease 2019 (COVID-19) safety precautions were adhered to, and the interaction with staff members was limited to a maximum of two individuals at a time. Participants were not paid to take part in the study; however, there was a lucky draw of one cash prize of R500 to incentivise participation.

### Data analysis

Categorical variables were summarised as counts and percentages and presented descriptively in frequency tables. Associations between questions and the predictor variable, academic qualification, were assessed using Chi-square tests at the 0.05 level of significance. Statistical analysis was conducted using the IBM SPSS Statistics version 27 software (IBM Corp., Armonk, NY, USA).

### Ethical considerations

Approval to conduct the study was obtained from Stellenbosch University Health Research Ethics Committee (S20/02/036), National Health Research Database (WC-202110328) and the TBH medical manager. This study was conducted in accordance with the 2013 Declaration of Helsinki (World Medical Association Declaration of Helsinki 2013:2191) and the South African Good Clinical Practice Guidelines (Department of Health, South African Good Clinical Practice 2020).

## Results

Sixty-eight RNs completed the questionnaire, giving a 100% response rate. Of the participants, 48 (70.6%) held a diploma and 20 (29.4%) held a degree. Characteristics of participants are shown in Table 1.

**TABLE 1:** Characteristics of participants.

Characteristics	Diploma		Degree		Total	
	*n* = 48	%	*n* = 20	%	*n* = 68	%
**Province of training**						
Eastern Cape	1	2.1	0	0	1	1.5
Free State	-	0	1	5.0	1	1.5
Gauteng	1	2.1	3	15.0	4	5.9
Kwazulu-Natal	4	8.3	0	0	4	5.9
Western Cape	37	77	16	80.0	53	77.9
Not specified	5	10.4	0	0	5	7.4
**Surgical nursing experience**						
< 5 years	14	29.2	9	45.0	23	33.8
5–10 years	7	14.6	1	5.0	8	11.8
> 10 years	27	56.3	10	50.0	37	54.4
Additional qualifications*	28	58.3	9	45.5	37	54.4

* , Post graduate diploma in: Administration; Critical Care; Education; Occupational Health; HIV & AIDS or Orthopedics & Trauma Emergency Care

Sixty-one (89.7%) participants knew the correct reason for keeping patients NPO. All 20 (100%) of the participants who held a degree answered correctly versus 41 (85.4%) from the diploma group. This difference was not statistically significant (*p* = 0.197). Of the participants, 65 (95.6%) answered yes to keeping patients NPO from midnight the night before surgery. The number of correct answers for the fasting times for food and various fluids and comparisons between the groups are shown in Table 2. There was a statistically significant difference in knowledge between diploma and degree participants for the non-human milk fasting time (*p* = 0.005), with more degree nurses answering correctly (Table 3).

**TABLE 2:** The number of correct answers for the fasting times for food and various fluids and comparisons between diploma and degree groups.

Food type	Diploma		Degree		Total		*p*-value
	*n* = 48	%	*n* = 20	%	*n* = 68	%	
Solids	45	93.8	20	100.0	65	95.6	0.52
Non-human milk	26	54.2	19	95.0	45	66.2	0.005*
Breast milk	21	43.8	11	55.0	32	47.1	0.116
Infant formula	18	37.5	12	60.0	30	44.1	0.228
Clear fuids	18	37.5	9	45.0	27	39.7	0.597

* , *p* < 0.05.

**TABLE 3:** Chi-square tests: Non-human milk.

	Value	*df*	Asymptotic sig. (2-sided)	Exact sig. (2-sided)	Exact sig. (1-sided)	Point probability
Pearson Chi square	10.789†	2	0.005	0.004	-	-
Likelihood ratio	14.597	2	0.001	0.001	-	-
Fisher-Freeman-Halton exact test	11.258	-	-	0.002	-	-
Linear-by-Linear Association	5.826^‡§^	1	0.016	0.015	0.006	0.004
Number of valid cases	68	-	-	-	-	-

sig., significance.

† , 2 cells (33.3%) have expected count less than 5;

‡ , the minimum expected count is 2.94;

§ , the standardised statistic is -2.414.

The practice of medication administration during the NPO period is shown in Table 4. Of the participants, 33 (48.5%) reported administering regular or chronic medication during the NPO period. Significantly more diploma than degree participants reported this practice (*p* = 0.037) (Table 5). Nineteen (27.9%) participants would allow patients to receive oral sips of water up to the time of surgery. There was no significant difference in this practice between diploma, 14 (29.2%) and degree, 5 (25.0%) participants (*p* = 0.588).

**TABLE 4:** The practice of medication administration during the NPO period.

Medication administered	Diploma		Degree		Total		*p*-value
	*n* = 48	%	*n* = 20	%	*n* = 68	%	
Oral analgesia	18	37.5	12	60.0	30	44.1	0.143
Intramuscular analgesia	46	95.8	18	90.0	64	94.1	0.352
Oral antibiotics	16	33.3	10	50.0	26	38.2	0.406
Intravenous antibiotics	47	97.9	19	95.0	66	97.1	0.517
Intravenous analgesia	45	93.8	16	80.0	61	89.7	0.115
Per rectum medication	30	62.5	15	75.0	45	66.2	0.523
Sublingual medication	26	54.2	11	55.0	37	54.4	0.159
Regular or chronic medication	28	58.3	5	25.0	33	48.5	0.037*

* , *p* < 0 05.

**TABLE 5:** Chi-square tests: Regular or chronic medication.

	Value	*df*	Asymptotic sig. (2-sided)	Exact sig. (2-sided)	Exact sig. (1-sided)	Point probability
Pearson Chi-square	6.596†	2	0.037	0.028	-	-
Likelihood ratio	6.787	2	0.034	0.032	-	-
Fisher-Freeman-Halton exact test	6.690	-	-	0.024	-	-
Linear-by-Linear Association	4.097^‡^	1	0.043	0.063	0.035	0.22
Number of valid cases	68	-	-	-	-	-

sig., significance.

† , 2 cells (33.3%) have expected count less than 5, the minimum expected count is 1.76;

‡ , the standardised statistic is 2.024.

## Discussion

Nursing personnel play a pivotal role in the multidisciplinary team involved with perioperative patient care. Fasting requirements are generally enforced by nurses and carried out in collaboration with patients. For this reason, it is essential that nurses possess up to date knowledge of preoperative fasting guidelines and medication administration during the NPO period.

Of the participants in this study, 89.7% knew the purpose of keeping patients NPO. This finding is in keeping with results from a survey performed in India in 2017 by Mohan et al. (2018:127) where 92.5% nurses knew the correct answer.

Participants were knowledgeable regarding fasting times for solids. In the study by Mohan et al. (2018:127), 20% of nurses correctly responded that a preoperative fasting time of 6 h for solids were required and 66.6% responded that solid food must be stopped 8 h before surgery. The participants knowledge of fasting times for breast milk and infant formula was poor. A possible explanation could be that the majority of participants in this study work in adult surgical wards.

The correct fasting time for clear fluids was answered correctly by 39.7% participants. This is of concern as thirst was shown in a qualitative study in 2012 by Carey et al. (2015:1946), to be the worst physical effect experienced by patients during the fasting period. In a study by McCraken and Montgomery (2018:37) in the United Kingdom, it was reported that the incidence of postoperative nausea and vomiting was reduced in patients who were allowed clear fluids up until the time of surgery. Also, reducing preoperative fasting times may improve patient satisfaction (Bopp et al. 2011:680; Imbelloni, Pombo & Filho 2015:117). A shortcoming of this study performed at TBH was that participants were not asked what constituted a clear fluid. This could be a possible explanation for the lack of knowledge in clear fluid fasting times in this study.

Despite 95.6% of participants knowing the correct fasting time for solids and 39.7% knowing the correct fasting time for clear fluids, 95.6% of participants reported that they would keep patients NPO from midnight the night before surgery. Of the participants in this study, 54.4% had qualified more than 10 years ago and knowledge obtained from their curriculum may now be outdated. Implementation of regular in-service training may ensure that knowledge remains current and up-to-date with best practices in medicine.

Parenterally administered medication during the NPO period showed good practice among both groups of participants. Only 44.1%, 38.2% and 48.5% participants, respectively, answered the questions about oral analgesia, oral antibiotics and chronic medication administration during the NPO period correctly. In contrast to the study by Mohan et al. (2018:127), 89.3% of nurses knew that chronic medication may be taken during the NPO period. In 2000, a study conducted by Kennedy et al. (2000:353) in New Zealand showed that withdrawal of regular medications may add significant risk to surgery and further complicate outcomes. The authors found that as the time without chronic medication increased, so did the complication rate. Of the patients dependent upon cardiovascular medication that went without their regular medication, 12% suffered cardiac complications (Kennedy et al. 2000:353). The lack of knowledge in participants in this study places patients at risk of complications from not receiving their chronic medications. An Australian study by Symons and McMurray (2014:267) in 2014 looked at the factors influencing nurses to withhold surgical patients’ oral medications perioperatively. Three main themes emerged from this study, namely nurses’ perceptions of their roles, ward culture and various patient factors (Symons & McMurray 2014:267). Ward culture regarding outdated practices and nurses’ perceptions of their role in the multidisciplinary team may be contributing factors to the findings in the TBH study.

### Limitations

This study was limited to one tertiary hospital in SA. As convenience sampling was employed, demographic distribution within this study setting may not be representative of the overall general study population of nursing personnel caring for surgical patients. Management of preoperative patients is a multidisciplinary team approach, and this study does not include knowledge and practice of other team members.

## Recommendations

It is proposed that quality assurance programmes addressing the findings of this study be implemented. A follow-up study after implementation could be conducted to assess the effectiveness of this intervention and assist in how guidelines could be implemented as current practice is not in the best interest of patients.

## Conclusion

Knowledge of fasting guidelines is an essential component of nursing care in the preoperative period. Knowledge of fasting times for solids and for parenterally administered medication during the preoperative period was adequate. Of concern was the lack of knowledge for fasting times for various fluids. In addition, it was also observed that poor practice of medication administration for chronic medication and other orally administered medication existed. Education of RNs by common interest groups is needed to ensure that patients receive the most up-to-date evidence-based care during the NPO period.
